# Phase 0 Radiopharmaceutical–Agent Clinical Development

**DOI:** 10.3389/fonc.2020.01310

**Published:** 2020-08-18

**Authors:** Charles A. Kunos, Larry V. Rubinstein, Jacek Capala, Michael A. McDonald

**Affiliations:** ^1^Cancer Therapy Evaluation Program, National Cancer Institute, Bethesda, MD, United States; ^2^Radiation Research Program, National Cancer Institute, Bethesda, MD, United States; ^3^Cancer Imaging Program, National Cancer Institute, Bethesda, MD, United States

**Keywords:** radiopharmaceutical, phase 0 clinical trial, cancer, national cancer institute (NCI), radiotherapy

## Abstract

The evaluation of antibody-targeted or peptide-targeted radiopharmaceuticals as monotherapy or in oncological drug combinations requires programmatic collaboration within the National Cancer Institute (NCI) clinical trial enterprise. Phase 0 trials provide a flexible research platform for the study of radiopharmaceutical–drug pharmacokinetics, radiation dosimetry, biomarkers of DNA damage response modulation, and pharmacodynamic benchmarks predictive of therapeutic success. In this article, we discuss a phase 0 clinical development approach for human antibody-targeted or peptide-targeted radiopharmaceutical–agent combinations. We expect that early-phase radiopharmaceutical–agent combination trials will become a more tactical and more prevalent part of radiopharmaceutical clinical development in the near-term future for the NCI Cancer Therapy Evaluation Program.

## Introduction

A transition away from non-specific cytotoxic drugs or extended-field radiotherapy to use of targeted drugs or radiopharmaceuticals demands a reevaluation of the United States National Cancer Institute (NCI) clinical development strategy. The troublesome issues that undermine a conventional approach to clinical development are (a) high costs in patient, financial, or professional resources; (b) increasing complexity of research objectives in clinical trials; and (c) a natural belief that tolerable investigational agent toxicity begets efficacy (1, 2). An early phase I trial therefore sets as the primary objective the determination of the highest investigational agent dose that associates with tolerable toxicity [i.e., maximum tolerated dose (MTD)], which is then carried forward into phase II efficacy trials (3). In a phase II efficacy trial, objective tumor shrinkage (i.e., response rate) in single-arm trials (4) or protracted progression-free survival (PFS) or overall survival (OS) in randomized trials (5) determines the appropriateness for definitive randomized phase III trials. Randomized phase III trials are the gold standard method to isolate benefits from new treatment effects vs. conventional therapy effects.

For antibody-targeted or peptide-targeted radiopharmaceutical and oncological drug combinations, the determination of a biologically effective dose instead of an MTD might be the most relevant aim of an early-phase trial, even though both approaches are reasonable (Table 1). The development and implementation of sophisticated pharmacokinetic and pharmacodynamic tools in radiopharmaceutical clinical trials have been underutilized over the past four decades (6). Because of an ever-expanding number of antibody-targeted or peptide-targeted new molecular entities (NMEs), the resources needed for pharmacokinetic and pharmacodynamic study for each and every NME identified are not readily accessible to many cancer treatment investigators. But the NCI is in a favorable position to create and to develop such resources in the near-term and in the long-term (7). For example, the NCI Small Business Innovation Research Program (SBIR) grants discovery-phase projects aimed at the commercial development of radiopharmaceutical dosimetry-based tools for individual patient treatment planning (8). Routine accessibility of predictive pharmacodynamic biomarkers for early-phase trials would bring forth a more sophisticated development strategy for radiopharmaceutical–agent combinations.

**Table 1:** Differences between phase I and phase 0 trials.

**Variable**	**Phase I trials**	**Phase 0 trials**
Primary endpoint	Establish the recommended phase II dose	Establish the dose for target modulation
Dose escalation	Determine safety and adverse events	Achieve desired exposure for target modulation
Preclinical biomarker study	Not consistently performed before the trial	Required pharmacodynamic assay pretrial
Biomarker assay	Not consistently performed before the trial	Required pharmacodynamic assay integrated
Number for accrual	18 or more patients often	8–10 subjects
Dosing	Multiple	One or limited
Therapeutic benefit	None but exceptional responders occur	None expected
Tumor biopsies	Optional or limited	Mandatory and serial for target modulation
Pharmacokinetics	Batched and analyzed later	Real-time or near real-time

**Figure 1:**
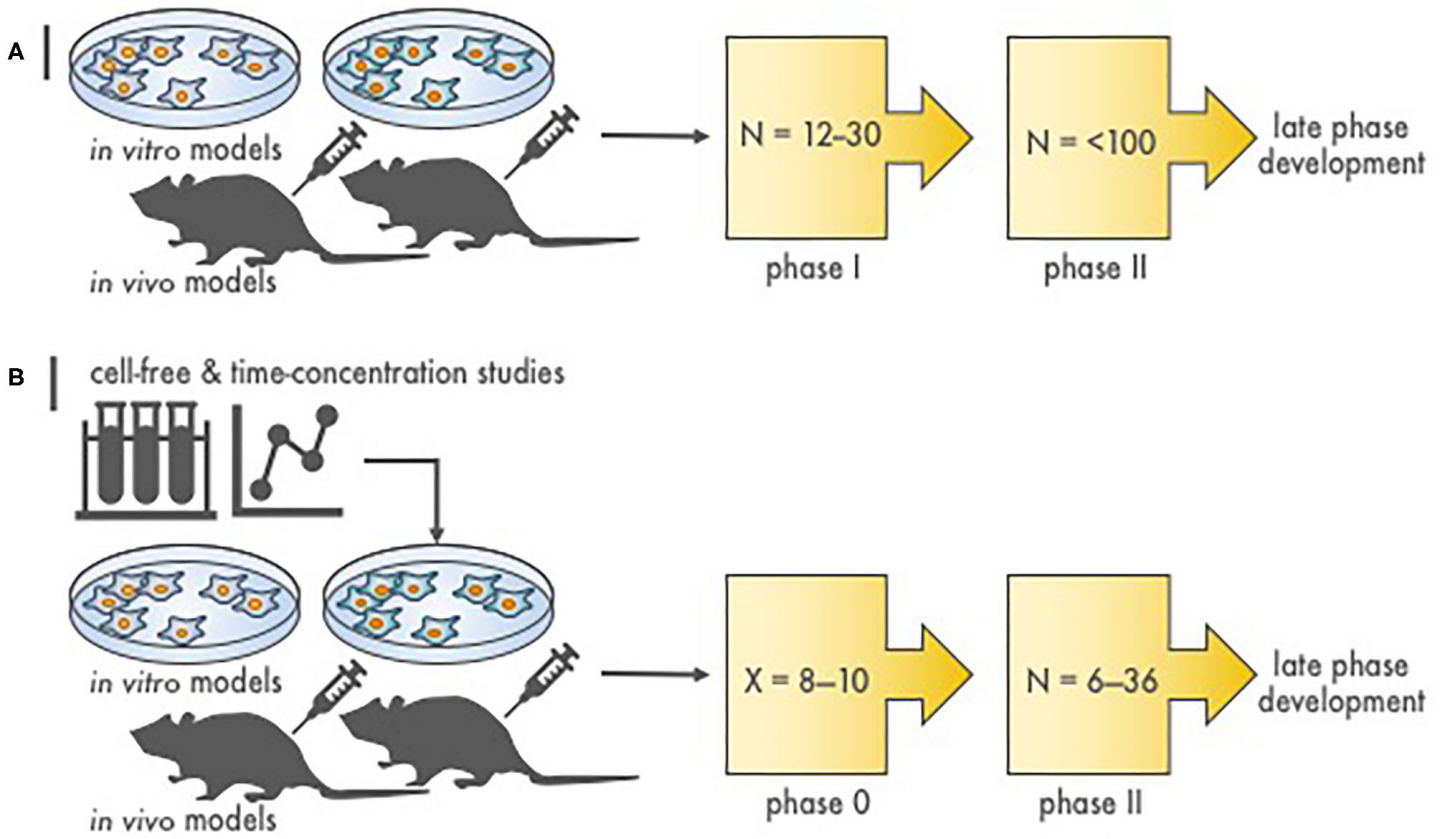
Stages of radiopharmaceutical–drug development. **(A)** Depicted are the steps to assess molecular target effects or cytotoxicity of a novel radiopharmaceutical–agent combination. N is the approximate patient sample size necessary to finish the phase of study. Proof-of-concept *in vitro* and *in vivo* experiments provide toxicity and efficacy endpoints, most often in two or more disease of interest models, that justify conventional phase I and II testing. **(B)** Illustrated are the stages to assess molecular target effects or cytotoxicity of a novel radiopharmaceutical–agent combination utilizing a compressed phase 0 approach. X is the estimated number of subjects required to complete a phase 0 study (~8–10). Proof-of-concept *in silico* or *first-in-human* microdosimetry studies (i.e., time-concentration studies) provide data that guide the planning and execution *in vitro* and *in vivo* in two or more disease of interest models. What follows is a phase 0 trial (pre-phase II trial) in a small number of subjects that use either single or shortened courses of radiopharmaceutical–agent treatment. This type of “target assessment” trial collects not only safety data but also definitive pharmacokinetic parameters, pharmacodynamic endpoints, and tumor responses in subjects with various cancer types. A phase 0 trial might provide a preliminary evaluation of whether irradiation or target engagement associates with clinical endpoints (i.e., tumor response). Phase 0 data inform statistical designs of “target validation” phase II efficacy trials by reducing patient numbers.

In the current NCI development strategy, after appropriate cancer-relevant preclinical experiments, phase I safety trials precede phase II efficacy studies, and then if justified, randomized phase III trials are conducted to compare a new agent combination to standard therapy (Figure 1). We propose that a radiopharmaceutical–agent combination development timeline could be shortened by the implementation of phase 0 trials that integrate pharmacokinetic and pharmacodynamic assessments to inform and to expedite next-phase development (Figure 1). At present, NCI Cancer Therapy Evaluation Program (CTEP) phase 0 trials are performed under an Exploratory Investigational New Drug (xIND) Application, as outlined in a 2006 Food and Drug Administration (FDA) guidance (9). We contend that the integration of pharmacokinetic and pharmacodynamic assays aids the evaluation of the radiopharmaceutical–agent (a) biological effects, (b) starting doses, and (c) schedules (Table 1). Phase 0 trials might also inform patient selection or response evaluations in subsequent phase II trials in a manner that typical phase I trials do (Table 1). This perspective is illustrated best by our thoughts on the FDA-approved somatostatin receptor-targeted lutetium-177 (^177^Lu) dotatate (Lutathera) that is intended for combination trials (10, 11). The challenges and opportunities within a therapeutic radiopharmaceutical–agent development strategy are discussed next in the context of ^177^Lu-dotatate clinical use.

## Challenges and Opportunities

A balance between *in vivo* peptide receptor production, trafficking, and subsequent degradation determines the quantified levels of those peptides utilized as biomarkers for drug effect. The antiproliferative action of the five-member seven-transmembrane domain G protein-coupled receptor superfamily for somatostatin illustrates this point (12–15). There are two biologic forms of somatostatin (i.e.,−14 and−18) that have variable affinity for the somatostatin receptors (SSTRs)—somatostatin-14 has highest affinity for SSTR1 through SSTR4, while somatostatin-18 binds selectively to SSTR5 (12). Four receptors (SSTR1, 2, 4, and 5) bring about cell cycle arrest either by an SHP-1/2-mediated or by a pertussis toxin-sensitive K+ channel-mediated inhibition of the Ras-Raf-Src-mitogen-activated protein kinase kinase (MEK) peptide cascade (16–19). Furthermore, it has also been shown that SSTR2 activates SHP-1 to upregulate the cell cycle regulator p27/Kip1, which sequesters Cdk2 and blocks cyclin E/Cdk2 complexing at the G1/S restriction checkpoint (20, 21). The SSTR2-SHP-1-p27/kip1 axis is particularly relevant to therapeutic anticancer strategies. SSTR3 uniquely triggers both SHP-2 to inactivate Raf (22) and SHP-1 for p53/Bcl-2 signal regulation of apoptosis (23). Also, SSTR3-mediated cell acidification renders caspase-8-mediated apoptosis (24). The structural analogs similar to somatostatin used in the medical clinic, octreotide and lanreotide, bind with highest affinity to SSTR4 and modest affinity to SSTR3 and to SSTR5 (25).

A therapeutic challenge arising from the production, trafficking, and degradation cycle of peptide receptors is that trial-ready pharmacodynamic studies might need the development and validation of up to three assays to grasp conclusions about therapeutic activity. To explain this point better, consider that if one patient had overexpression (high production) of targeted peptide receptors but low degradation, a pharmacodynamic microdose assay for a peptide-targeted radiopharmaceutical might predict that the patient is a responder. Consider that a different patient might have both high overexpression and high degradation of targeted peptide receptors. A pharmacodynamic microdose assay in this latter patient might predict response when there actually might not be one due to high degradation of targeted peptide receptors. For both patient scenarios, treatment response assessment might fall into a mixed, stable, or no response category. This sort of interpretive challenge influences calculation of a personalized radiopharmaceutical dose like for ^177^Lu-dotatate. Further study is warranted.

The chelator DOTA (tetraazacyclododecanetetraacetic acid)-Tyr^3^-octreotate (dotatate), the targeting peptide for the ^177^Lu radioactive payload, binds with greater affinity to SSTR2 than octreotide and thus has higher tumor surface bond (26, 27). Labeling dotatate by gallium-68 (^68^Ga) enables positron emission tomography-based diagnostic and microdosing capacity with improved sensitivity and specificity (28). It alone might not predict ^177^Lu-dotatate treatment response; this requires further research. ^68^Ga dotatate positron emission tomography enables the calculation of a personalized radiopharmaceutical dose (29). Therapeutic intent labeling of dotatate with ^177^Lu might therefore involve (a) a proportion bound to the targeted surface receptor (here, SSTR2), (b) a proportion internalized by receptor-mediated endocytosis that carries the radioactive ^177^Lu payload into the cell (30), and (c) a proportion that leads to high radioisotope concentration within the cancer cell after receptor degradation. In clinical studies, it is difficult to isolate whether surface, internalized, or intracellular localization contributes to objective response rates. Three clinical studies used this rationale for clinical development of the agent.

The first clinical study was conducted in Rotterdam, Netherlands, between 2000 and 2006 and enrolled 504 patients with ^111^In-DTPA octreotide scintigraphy-positive tumors of carcinoid, pancreatic neuroendocrine, and neuroendocrine of unknown origin (ERASMUS) (31). Patients received up to a ^177^Lu-dotatate cumulative dose of 750–800 mCi (27.8–29.6 GBq) intravenously divided in four 8-week cycles of ~200 mCi, which corresponded to a radiation dose to the bone marrow of 2 Gy, unless kidney dosimetry indicated that the radiation dose would exceed 23 Gy, and in these cases, the cumulative dose was reduced to 500–700 mCi. Antiemetics were injected intravenously before the start of the radiopharmaceutical. An infusion of amino acids (lysine 2.5%, arginine 2.5% in 1 L 0.9% NaCl; 250 ml/h) was started 30 min before the radiopharmaceutical and lasted 4 h. The objective response rate was 46% (31). Median progression-free survival and overall survival were 33 months and 46 months, respectively (31).

The first American multicenter single-arm trial experience of ^177^Lu-dotatate recruited 37 relapsed or refractory patients with ^111^In-DTPA octreotide scintigraphy-positive gastroenteropancreatic neuroendocrine tumors between 2010 and 2013 (32). Patients received up to four infusions of 200 mCi (7.4 GBq) ^177^Lu-dotatate every 8 weeks [cumulative dose 800 mCi (29.6 GBq)]. A 15% Clinisol amino acid solution (1 L) for renal protection was started 30 min before the radiopharmaceutical and lasted 4 h. Antiemetics were allowed. Patients were released from the treatment site when radiation exposure measured at 1 m at discharge was three to six millirem per hour (32). Eighty percent of patients administered at least one dose noted reversible nausea or vomiting; no grade 4 or higher toxicities were encountered. Thirty-one percent (10 of 32) had a response (32).

Between 2012 and 2016, the third clinical study was performed in 229 patients with inoperable well-differentiated (Ki67 index of 20% or less) somatostatin receptor scintigraphy-positive midgut neuroendocrine tumors and had measurable disease progression during treatment with octreotide long-acting repeatable (LAR) within a maximum of 3 years before enrollment (33). One hundred ten (98%) of 113 received high-dose octreotide LAR at a dose of 60 mg repeated every 4 weeks (control group). One hundred eleven (96%) of 116 received four infusions of 200 mCi (7.4 GBq) ^177^Lu-dotatate (experimental group) every 8 weeks [cumulative dose 800 mCi (29.6 GBq)]. For renal protection, intravenous amino acids [Aminosyn II 10% (21.0 g of lysine and 20.4 g of arginine in 2 L of solution) or VAMIN-18 (18 g of lysine and 22.6 g of arginine in 2 L of solution)] was started 30 min before the radiopharmaceutical and lasted 4 h. Octreotide injections were allowed in both treatment groups for hormonal symptoms (e.g., diarrhea or flushing). The objective response rate was 18% after ^177^Lu-dotatate and 3% after high-dose octreotide LAR (33). Median progression-free survival had not yet been reached after ^177^lutetium dotatate and was 8 months after high-dose octreotide LAR (33). For a 20-month progression-free survival estimate, ^177^Lu-dotatate resulted in 65% progression-free vs. 11% after high-dose octreotide LAR (33). At 20 months, an estimate of overall survival was 82% after ^177^Lu-dotatate and 50% after high-dose octreotide LAR, achieving a significant hazard ratio of 0.40 (*P* = 0.004; 33).

## Perspectives on Phase 0 Radiopharmaceutical Clinical Development

**Figure 2:**
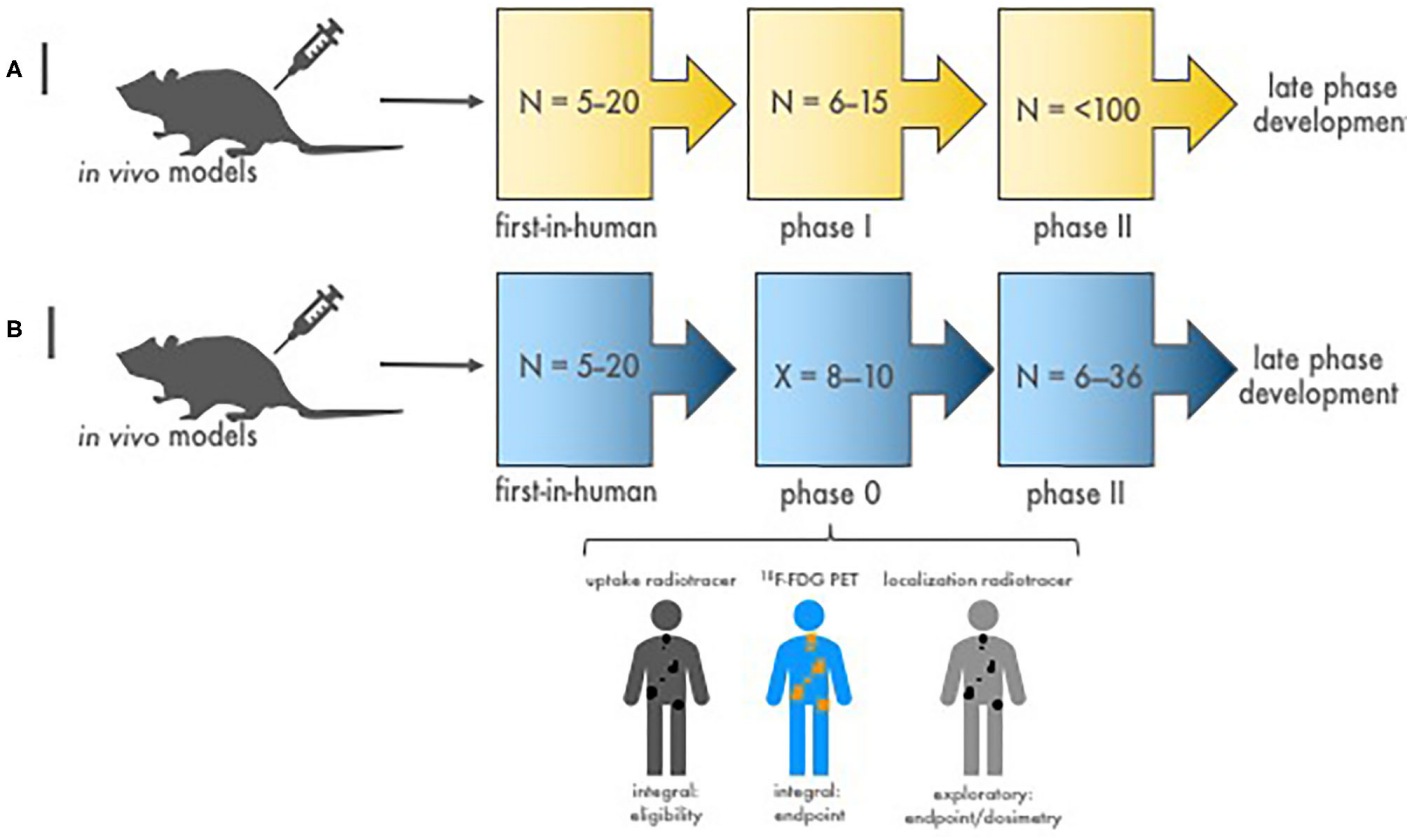
Stages of diagnostic-therapeutic or “theranostic” radiopharmaceutical development. **(A)** Illustrated are the conventional stages of early-phase development of diagnostic–therapeutic radiopharmaceutical pairs [like ^68^Ga (diagnostic) and ^177^Lu (therapeutic) for neuroendocrine cancers]. N is the estimated patient sample size needed to complete each study phase. Proof-of-concept *first-in-human* microdosimetry studies (i.e., time-concentration studies) characterize the initial relationship between antibody-receptor or peptide-receptor ligands using a diagnostic radionuclide (^68^Ga, in this example). Then, phase I patients enrolled with tumors shown to have diagnostic ligand positivity (^68^Ga retention on nuclear medicine imaging) are given therapeutic doses (^177^Lu, in this example) with or without oncologic drugs to evaluate the safety of treatment. Efficacy phase II trials are conducted to study clinical endpoints (i.e., tumor response, duration of response, and progression-free or overall survivals). If warranted, definitive phase III trials are done in late-phase development to compare the new treatment to standard treatment. **(B)** Depicted are the stages of diagnostic–therapeutic radiopharmaceutical pair development engaging a timeline-compressed phase 0 approach. N is the number of patients needed to complete the trial phase. X is the number of phase 0 subjects required for safety, pharmacokinetic, and pharmacodynamic endpoints (~8–10). The phase 0 trial might collect data on (a) a diagnostic radionuclide (i.e., an uptake radiotracer, ^68^Ga-dotatate) to demonstrate target positivity integral for trial eligibility before giving a therapeutic dose of an investigational radiopharmaceutical, (b) a conventional response indicator [like ^18^F-FDG positron emission tomography (PET)] as an integral clinical response endpoint assessment, and (c) a dosimetry radionuclide (i.e., localization radiotracer) to gauge actual irradiation dose in targeted tumors. Efficacy phase II trials are then conducted with a focused diagnostic–therapeutic radiopharmaceutical response with dosimetry substudies. If promising, a definitive phase III trial follows to contrast clinical endpoints after new or standard treatments.

**Figure 3:**
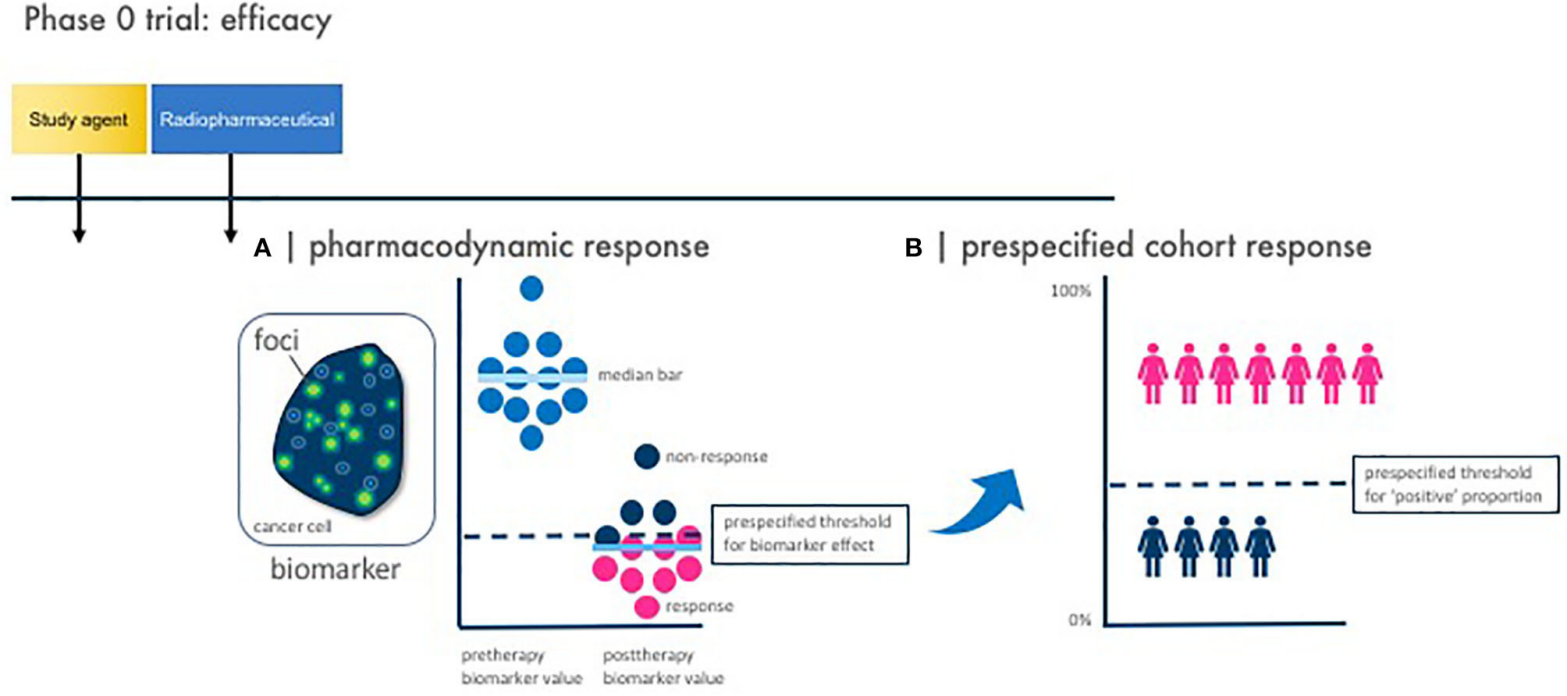
Phase 0 trial pharmacodynamic efficacy endpoints. Illustrated here are the two vital study design considerations for a phase 0 trial with pharmacodynamic efficacy endpoints. Baseline and posttherapy biomarker assessments are obtained for pharmacodynamic response. Response is defined by two parameters—a pharmacodynamic response and a prespecified cohort response. **(A)** A pharmacodynamic response is scored positive when a biomarker signal [like γH2AX foci immunofluorescence area (green dots)] passes a prespecified threshold for biomarker effect. **(B)** A prespecified cohort response is scored positive when the number of subjects showing a positive pharmacodynamic response passes a prespecified threshold for “positive” proportion. This two-step process defines what establishes a favorable observed pharmacodynamic response rate in the phase 0 trial—in other words, how many subjects must demonstrate a pharmacodynamic response for the phase 0 trial to be declared biologically effective. This is parallel to determining a threshold for observed response rate in a phase II trial in order that the radiopharmaceutical–agent combination be considered sufficiently favorable for further testing in trials.

Crucial inquiries in conventional agent development are whether dose and schedule of an agent combination impacts efficacy. *One* such approach among many alternatives is to use a phase 0 trial of a single optimal dose or a limited number of repeated doses in a variety of schedules with pharmacokinetic and pharmacodynamic evaluations (Figures 1–3). Pharmacodynamic evaluations might use blood-based assays that inspect the level of DNA damage marked by γH2AX foci in lymphocytes produced by an in-transit radiopharmaceutical–agent combination (34–36). The optimal schedule and sequence to use in agent combination studies might be judged as the one that optimal levels of DNA damage in lymphocytes by the addition of the radiopharmaceutical–agent pair corresponds to a predetermined threshold for therapeutic tumor response or “success.” Other sources for γH2AX foci change could be skin hair follicles. For such combinations, the combinatorial impact of one radiopharmaceutical on another oncology agent might occur at radiation prescription doses well-below the traditional oncology agent maximum tolerated dose. We contend that without adequate pharmacodynamic testing, a chance for optimized phase II trial design is vacated. Any pharmacokinetic retention or organ elimination data for different administered dosings would inform investigators as to whether adverse events of special interest should be monitored in future trials. The pharmacokinetic data would also allow estimation of the radiation dosimetry (or irradiation dose delivered to the tumor and normal organs of risk like the kidneys and bone marrow). A phase 0 trial approach evaluating a small number of doses and schedules involving a limited number of subjects might speculatively advise next-step trials (Figures 1–3).

**Figure 4:**
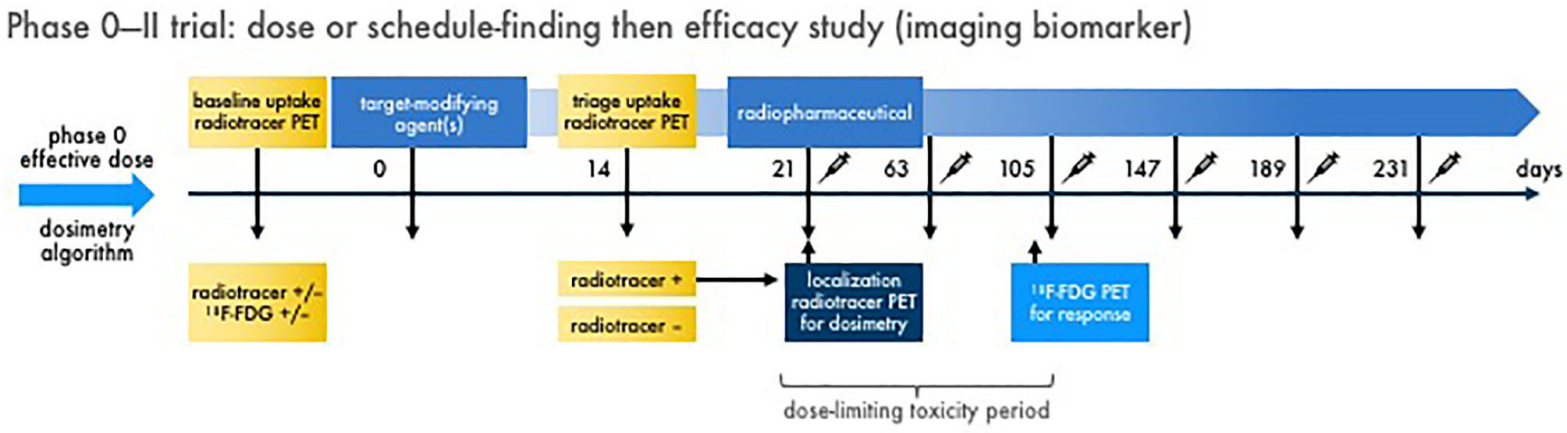
Multiple dose radiopharmaceutical–agent combination phase 0–II trial with imaging endpoints. Schemed here are the elements for one example of a phase 0 dose or schedule-finding trial transitioning to a phase II efficacy trial with imaging biomarkers. Figure 2 discusses the phase 0 trial approach. In phase II, baseline diagnostic imaging (like an uptake radiotracer, ^68^Ga-dotatate) and conventional response indicator [like ^18^F-FDG positron emission tomography (PET)] is acquired for reference. A target-modifying agent (or drug) is given, and then repeat diagnostic uptake radiotracer imaging is acquired to triage patients with “positive” tumors forward to therapeutic radiopharmaceutical treatment. On the day of radiopharmaceutical delivery, a dosimetry substudy [like a single photon emitted computed tomography (SPECT) scan for ^177^Lu-dotatate] is done for the purpose of calculating actual irradiation dose in targeted tumors. What follows are multiple administrations of radiopharmaceutical–agent combination treatments in prespecified doses and schedules. A defined dose-limiting toxicity observation window (for up to two cycles to capture “late” adverse events) is used for safety endpoints. The conventional response indicator performed at baseline is repeated (like after two cycles) for response assessment. Compelling results from a phase 0–II trial approach might lead to definitive phase III trials. It is important to note that links or discussion of this radiopharmaceutical–agent phase 0–II trial design does not constitute endorsement nor commit the US Federal government to this approach.

Therapeutic radiopharmaceuticals are highly specific, have desirable in-residence time at the target, and have favorable elimination characteristics that ensures optimal tumor to background differentiation. Diagnostic-therapeutic radiopharmaceutical pairs, so-called “theranostics,” might be evaluated using microdose studies recruiting a small number of phase 0 subjects to study biodistribution, in-residence time, radiation dosimetry, and corresponding biologic effect. In this way, a radiopharmaceutical–agent phase 0 trial might triage patient populations for future next-phase trials. Take for example the radiopharmaceutical–imaging agent pair of ^177^Lu-dotatate and ^68^Ga-dotatate (37–39). Figure 4 depicts concepts surrounding the parameters of a phase II trial predicated on the findings of a lead-in phase 0 trial. In some cases, an agent or drug might modify the antigen target that an antibody-targeted or peptide-targeted radiopharmaceutical depends. An agent or drug alone window of exposure might be important for determining the efficacy of a radiopharmaceutical–agent pair. Certainly, the length of this window varies by pharmacokinetic factors and biologic responses. Reimaging to ensure tumor “positivity” after and agent or drug alone window is reasonable to ensure radiopharmaceutical targeting. Dosimetry-based scans are done to determine irradiation dose delivered (and might vary according to emitted particle [i.e., alpha particle, beta particle, or conversion electron] and decayed particle penetrance in tissue (e.g., ^223^radium-emitted alpha particle range = 40 μM or 10 cell diameters; ^177^lutetium-emitted beta particle range = 350 μM or 27 cell diameters) (40). In this example, ^68^gallium-dotatate site intensity relative to normal tissue background can be used to determine an individual patient's tumor burden, target in-residence time, and tumor heterogeneity so that subsequent calculation of therapeutic radiopharmaceutical dose could be optimized for maximal tolerated radiation dose to tumor burden without undue harm to normal organs at risk (40). In traditional radiopharmaceutical–agent combination discovery, decisions about lead therapeutic agent selection for further development are made on the basis of *in vitro* and *in vivo* animal model data, which is difficult to do for oncologic radiopharmaceutical agents because of radioisotope handling. Owing to limited financial, patient, and professional resources, early-phase radiopharmaceutical safety and efficacy studies underperform and might lead to promising combinations not being developed fully. We contend that radiopharmaceutical–agent early-phase trials that incorporate phase 0 trial elements will provide essential human pharmacokinetic and pharmacodynamic data that are informative to trial decision-making by stakeholders. Integrating phase 0 trial elements consistently and in the long-term will also establish guidelines for items in national coverage analyses, which currently might be blocks to discovery and development.

## Conclusion

In summary, this perspective article discusses the potential use of phase 0 trial elements as they relate to radiopharmaceutical–agent clinical development. It offers strategic insights into the interpretation of phase 0 trial biomarker response and predictions of therapeutic success. Education of both research subjects and their radiation oncologists or nuclear medicine physicians in the use of radiopharmaceuticals remains essential to the beneficial clinical development of these types of anticancer treatments.

## Data Availability Statement

The original contributions presented in the study are included in the article/supplementary material, further inquiries can be directed to the corresponding author/s.

## Author Contributions

CK, LR, JC, and MM contributed to the collection and review of any perspective data, analysis and authentication, the writing, and approval of this manuscript. All authors contributed to the article and approved the submitted version.

## Conflict of Interest

The authors declare that the research was conducted in the absence of any commercial or financial relationships that could be construed as a potential conflict of interest.
